# Treatment of Common Femoral Artery Pseudoaneurysm: A Novel Approach Utilizing a VASCADE Percutaneous Closure Device

**DOI:** 10.1155/2019/1397981

**Published:** 2019-05-27

**Authors:** Mark Dalvin, Brandon Dessecker, Eugene Vitvitsky

**Affiliations:** ^1^Western Reserve Health Education, USA; ^2^Trumbull Regional Medical Center, USA

## Abstract

Iatrogenic common femoral artery pseudoaneurysm is a well-known complication to vascular access. Many options, both surgical and nonsurgical, have been implemented as means to treat pseudoaneurysms such as thrombin injection, image-guided compression, and percutaneous closure devices. This case report demonstrates a novel technique using a VASCADE closure device to successfully treat an iatrogenic common femoral pseudoaneurysm.

## 1. Introduction

Common femoral pseudoaneurysms are a known complication to vascular access with an incidence ranging from 0.2 to 7% [1, 2]. Several treatment options have been utilized to avoid the risks of open surgery. Among these are ultrasound-guided compression, direct percutaneous thrombin injection, selective embolization via coils or n-butyl cyanoacrylate, stent placement, and saline/lidocaine injection with compression [3].

Of the above treatment options, thrombin injection and ultrasound-guided compression have been used more frequently and thus have more available study data. A Cochrane review suggests slight support of the use of thrombin injection over compression, but statistical significance is not present in total [4]. Percutaneous thrombin injection has been shown to be very successful with reports showing near 100% success rates [5]. However, it does come with a small risk of embolization, particularly with short-necked pseudoaneurysms, and failure warrants a different treatment option [6].

Previous case reports have shown success with a vascular closure device called Angio-Seal, which uses an endovascular bioabsorbable anchor and extravascular collagen sponge to seal and thrombose the pseudoaneurysm [7–9]. However, the use of a different vascular closure device, VASCADE, to treat a common femoral pseudoaneurysm has not been reported.

VASCADE is a vascular closure device made by Cardiva Medical that can be inserted into a 5 French, 6 French, or 7 French procedural sheath. It is a fully integrated system that boasts the ability to close a femoral access site without leaving any permanent components inside or outside the vessel. It uses a bioabsorbable extravascular collagen component to achieve hemostasis with the aid of compression from an expandable endovascular disc [10]. This method has been shown to be safe via the RESPECT trial. This was a randomized clinical trial showing a clinically significant reduction in minor vascular complications while maintaining a similar major vascular complication rate compared to manual compression [11].

This report shows a single-case success with the VASCADE closure device in treating an iatrogenic right common femoral artery pseudoaneurysm.

## 2. Case Report

**Figure 1:**
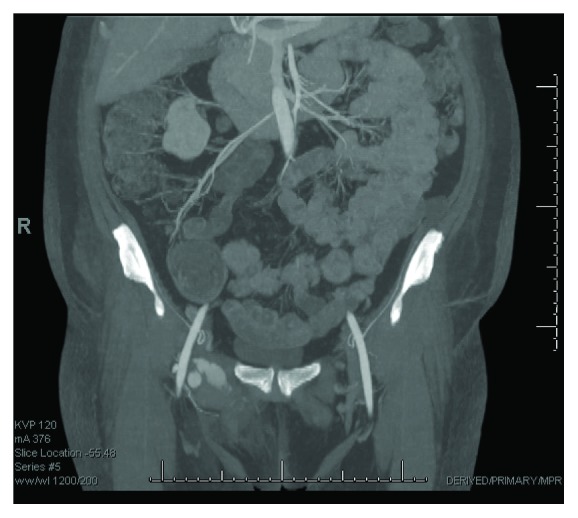
CTA showing right common femoral artery pseudoaneurysm.

The patient is a 57-year-old female who underwent cardiac catheterization via the right common femoral artery two weeks prior to developing a large, symptomatic right common femoral artery pseudoaneurysm (Figure 1).

The patient began complaining of groin pain two weeks after cardiac catheterization. She has a past medical history of aortic valve replacement secondary to aortic valve infective endocarditis, hyperlipidemia, and hypertension.

**Figure 2:**
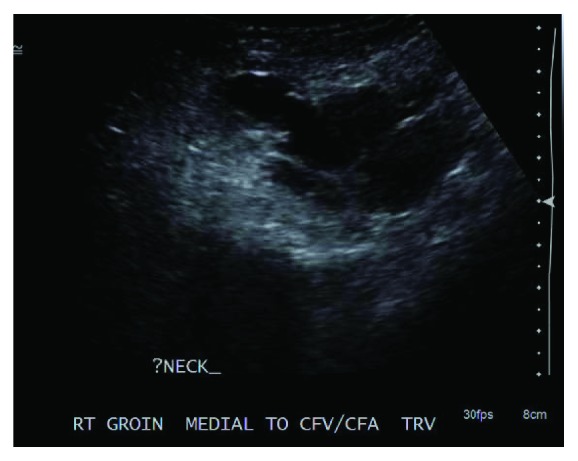
Duplex Ultrasound of the right common femoral artery pseudoaneurysm after the first thrombin injection.

**Figure 3:**
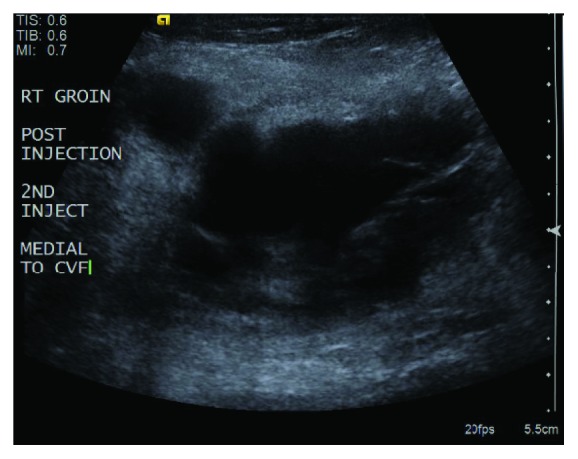
Duplex Ultrasound of the right common femoral artery pseudoaneurysm after the second thrombin injection.

She underwent two attempts of ultrasound-guided thrombin injection of the pseudoaneurysm. On ultrasound, the size of the pseudoaneurysm was found to be 5 cm × 3 cm × 4.6 cm. The neck of the pseudoaneurysm was measured to be 0.8 cm long. The two attempts involved using a 21 gauge needle to administer 1000 units and 2000 units of thrombin, respectively, into the pseudoaneurysm under ultrasound guidance and with the assistance of compression. Due to the size of the aneurysmal cavity and a relatively large pseudoaneurysm neck, injections were found to be unsuccessful on follow-up ultrasound (Figures 2 and 3). It was then decided to attempt endovascular closure of the neck of the pseudoaneurysm. All risks were discussed with the patient.

After identification by the attending surgeon, the patient was transferred to the procedure room table in the catheterization lab. The patient received IV sedation, and local anesthesia was used prior to ultrasound-guided percutaneous access to the left common femoral artery. During the procedure, vital signs, including blood pressure, heart rate, respiratory rate, and oxygen saturation, were monitored by an ACLS certified nurse.

**Figure 4:**
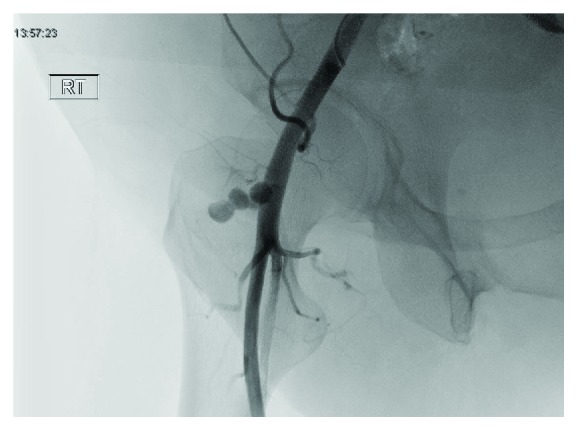
Visualization of pseudoaneurysm prior to the use of VASCADE.

After a 21 gauge needle was placed into the projection of the vessel lumen, a guidewire was placed into the left iliac artery. An angiographic catheter and guidewire were used to perform selective cannulation of the contralateral right common iliac artery. Then, a 6 French long access sheath was placed to perform an angiogram. The neck of the pseudoaneurysm was visualized (Figure 4), and a 0.014 guidewire was placed into the proximal portion of the neck.

**Figure 5:**
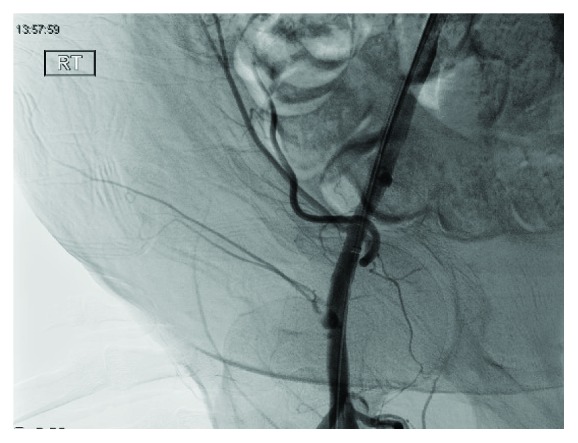
Partial occlusion of pseudoaneurysm after VASCADE closure.

A 21 gauge needle was used to cannulate the proximal portion of the neck percutaneously from the right groin. The previously placed guidewire was used as a landmark to place the tip of the 21 gauge needle into the pseudoaneurysm. After blood return was noticed from the needle, a 0.018 guidewire was placed into the lumen of the right common femoral artery. A 6 French access sheath was placed over the guidewire. Fluoroscopy was then used to visualize the deployment of a vessel closure device (VASCADE 6 French). This was done without difficulty, and the collagen patch was positioned outside the vessel wall in the area of the pseudoaneurysm neck. Interval angiogram revealed partial occlusion of the pseudoaneurysm neck (Figure 5).

**Figure 6:**
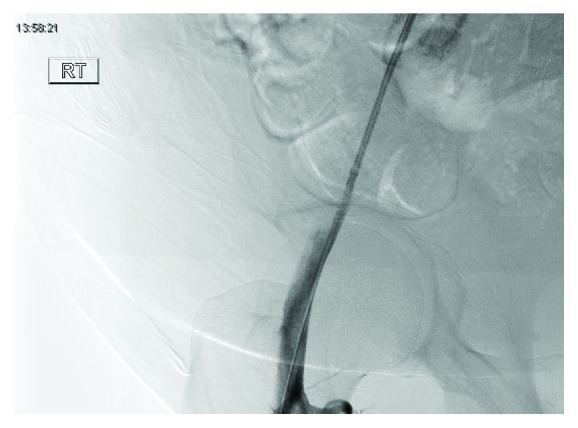
Image after the first balloon insufflation.

**Figure 7:**
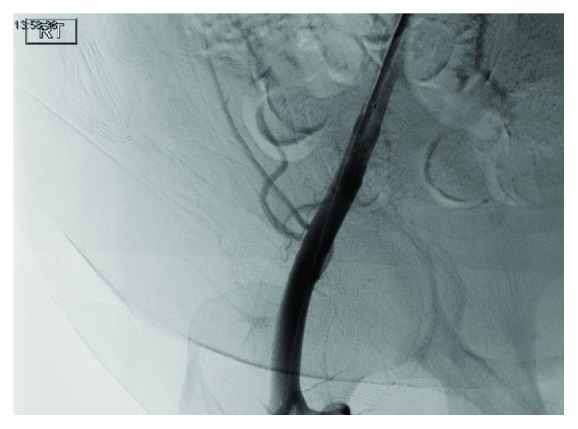
Image after the second balloon insufflation.

It was then decided to place an occlusive 8 mm balloon into the lumen of the right common femoral artery to facilitate pseudoaneurysm thrombosis. The balloon was insufflated up to 8 ATM for 600 seconds. This was done twice in total. Interval angiogram then revealed complete occlusion of the pseudoaneurysm blood flow (Figures 6 and 7).

All wires and catheters were removed at this point, and a left common femoral artery access sheath was kept in overnight. Postoperatively, the patient had no complications, and formal ultrasound confirmed complete thrombosis of the pseudoaneurysm. The access sheath was then removed without issue. There were no ischemic complications due to balloon occlusion in the immediate postoperative period.

## 3. Discussion

There is more data available exploring the efficacy of both manual compression and thrombin injections as means to treat common femoral artery pseudoaneurysms. These options are currently still recommended as earlier options than the use of vascular closure devices. However, this case report allows insight into additional options when first-line treatment fails. It also offers a suggestion for further data collection to explore the efficacy of this alternative.

While other case reports have shown successful closure of common femoral artery pseudoaneurysms with the use of Angio-Seal, the success of VASCADE may provide a superior alternative to novel treatments. Particularly, the lack of an endovascular component in VASCADE compared with Angio-Seal's endovascular anchor eliminates the concern for foreign body embolization. This complication has been documented to cause acute distal limb ischemia [12, 13]. Elimination of the risk of such a complication, while rare, suggests that exploration of VASCADE's efficacy in pseudoaneurysm treatment may be more appealing than studying Angio-Seal's efficacy. Likewise, there may be a similar advantage to VASCADE over using an endovascular stent, again due to the complications of endovascular components, particularly occlusion in the case of stents. There are no clinical trials assessing the success of endovascular stenting as a treatment for pseudoaneurysms. However, data from a multicenter peripheral interventional registry showed an overall 4.3% femoropopliteal stent thrombosis rate over a median follow-up interval of six months [14].

## 4. Conclusion

This case report highlights a novel treatment for iatrogenic common femoral artery pseudoaneurysm using the VASCADE closure device. This option may be useful when manual compression or thrombin injections fail, and it may be superior to Angio-Seal due to eliminating the need to leave an anchor within the artery. A more comprehensive study needs to be done in order to truly understand the efficacy of this treatment in pseudoaneurysm closure.
